# Miscellaneous Items

**Published:** 1866-02

**Authors:** D. L. Overholser

**Affiliations:** Morris, Ill.


### MISCELLANEOUS ITEMS.

By D. L. Overholser.

Wood's Alloy.—I commenced using this material for filling about two years ago. For a time I was much pleased with it, and used it somewhat extensively; but later I found quite a number of the fillings fail. Some would be loose, and still remain in the cavity, owing to its shape and position, others came out; and in some cavities, I found, to an alarming extent, that softened yellow dentine, of which Prof. J. D. White spoke in the "Dental Cosmos" for May, 1865. In view of these facts, I abandoned the material, after using it for about fifteen months. Since then, when I have to use plastic fillings for back teeth, I use Lawrence's Amalgam.

Lower Impression Cups.—Formerly I had very considerable trouble in taking impressions of the lower jaw. The principal cause of the trouble was long impression cups, No. 3 being the smallest I then had. I got a No. 4, (White's), and don't remember having used any but that since. I have a No. 2 cup, but unless I should take it into my mind to experiment in veterinary dentistry, I don't expect ever to find any use for it. It can, however, easily be made useful by cutting off the ends.

Another Vulcanizer Explosion.—I noticed, several weeks ago, in a Chicago daily paper, that another vulcanizer had exploded in that city. This time, as in other cases of the kind, the person connected with it is said to have had a narrow escape from death. The account said nothing of the kind of vulcanizer nor of the cause of the explosion. These are points of special interest to those who are constantly using these instruments. I hope Dr. Albaugh, in whose office it is said to have occurred, or some one else familiar with the circumstances of the case, will enlighten the profession through the dental periodicals.

INFORMATION WANTED.— I have had serious trouble in attempting to supply several soft mouths with artificial upper teeth. The softness was not extreme, nor did it extend, in any considerable degree, to the alveolor ridge, which was thick. The so-called hard palate was soft, especially on each side of the ridge of the palate. The softness was not of a spongy character, but apparently muscular. For the last case of the kind I had, I made two plates (rubber), both of which apparently fitted; but although they could be worn some, and were worn, as I was assured perseveringly, for several months, yet they adhered very slightly, and dropped down from very slight causes. I would like to get the experience of others with cases of this kind, and if any one will, through the Register, tell me of a mode by which such cases can be treated successfully, I will be greatly obliged. If there should be no response to this I shall conclude that among all the readers of the Register, there is not one who succeeds any better than myself, or, if there is one, his intense selfishness renders him unworthy a membership in the dental profession of the present age.

I would here suggest that a department in the Register, of questions and answers, on various dental topics, could be made exceedingly useful and interesting, if the profession, generally, would engage in it. Shall we have it?

